# Optical coherence tomography angiography characteristics of choroidal neovascularization requiring varied dosing frequencies in treat-and-extend management: An analysis of the AVATAR study

**DOI:** 10.1371/journal.pone.0218889

**Published:** 2019-06-25

**Authors:** Atsuro Uchida, Ming Hu, Amy Babiuch, Sunil K. Srivastava, Rishi P. Singh, Peter K. Kaiser, Katherine Talcott, Aleksandra Rachitskaya, Justis P. Ehlers

**Affiliations:** 1 The Tony and Leona Campane Center for Excellence in Image-guided Surgery and Advanced Imaging Research, Cole Eye Institute, Cleveland Clinic, Cleveland, Ohio, United States of America; 2 Department of Quantitative Health Sciences, Lerner Research Institute, Cleveland Clinic, Cleveland, Ohio, United States of America; 3 Retina Service, Cole Eye Institute, Cleveland Clinic, Cleveland, Ohio, United States of America; Massachusetts Eye & Ear Infirmary, Harvard Medical School, UNITED STATES

## Abstract

**Purpose:**

To evaluate optical coherence tomography angiography (OCTA) characteristics of choroidal neovascularization (CNV) in eyes requiring different treatment frequency of anti-vascular endothelial growth factor (VEGF) therapy for neovascular age-related macular degeneration (NVAMD).

**Design:**

Prospective observational case series.

**Methods:**

Subjects who had undergone anti-VEGF treatment for NVAMD in the AVATAR study were subdivided into 3 groups depending on required anti-VEGF dosing: (i) treat-and-extend requiring every 4–6 weeks dosing (TEq4-6w), (ii) treat-and-extend requiring every 7–12 weeks dosing (TEq7-12w), (iii) eyes not requiring injection within last 12 months (PRN >12mo). OCTA images were evaluated for the morphological characteristics of CNV and the choriocapillaris flow void.

**Results:**

Study consisted 40 eyes of 31 patients with a mean age of 79.9 ± 6.2 years. CNV morphology analysis on OCTA was feasible in 29 (73%) eyes. Ninety percent of CNVs in TEq7-12w group were irregular in shape involving foveal center, while 67% of CNVs in PRN>12mo group were circular in shape sparing foveal center. Among three groups, statistical difference was found in CNV shape (P *=* .012) and CNV location (P *=* .003), while no statistical difference was found in the CNV area (P *=* .14), vessel density (P *=* .19), presence of core vessels (P *=* .23), the presence of small margin loops (P *=* .20), large margin loops (P *=* .14), CNV maturity (P *=* .40), or the mean percentage of choriocapillaris area with flow void (P *=* .66).

**Conclusion:**

The combination of CNV sparing the foveal center with higher circularity may suggest a clinically inactive CNV following initial anti-VEGF therapy. We found minimal distinguishing OCTA characteristics between those eyes that required ongoing therapy with the treat-and-extend regimen. More research is needed to identify specific CNV characteristics on OCTA that may become a useful tool for the management of NVAMD and timing of treatment.

## Introduction

Neovascular age-related macular degeneration (NVAMD) is one of the leading causes of visual impairment and blindness among elderly individuals in the developed world [1]. Over the last decade, anti-vascular endothelial growth factor (VEGF) therapy has significantly reduced the risk of vision loss and has become the mainstay of treatment for NVAMD [2–4]. Given that therapeutic responses vary among individuals [5, 6] and long-term ongoing intravitreal injections are often required to maintain the therapeutic effect [5, 7], the optimization of treatment interval has a crucial role in minimizing patient’s treatment burden. Currently, many physicians favor an individualized approach to dosing frequency, utilizing either a “treat-and-extend (T&E)” or a “treat as-needed (PRN)” regimen [8], as these dosing approaches can reduce the chance of overtreatment (the number of injections) while achieving similar therapeutic benefits compared with monthly dosing regimen [9, 10]. The cause of variable therapeutic requirement to ant-VEGF therapy is not fully established. However, it is potentially related to factors such as age [11], genetic background [12, 13], drug selection [14], tachyphylaxis development [15, 16], type of choroidal neovascularization (CNV) [17], CNV size [11] and CNV maturity [18, 19].

Recently introduced OCT angiography (OCTA) has enabled non-invasive visualization and delineation of flow signals within CNV in NVAMD [20–22]. Previous studies have described various effects of anti-VEGF therapy on the flow signal within CNV, including a reduction in size, complete flow resolution, and persistence of flow [23–26]. However, there has been little exploration on evaluating the characteristics of CNV on OCTA following establishment of optimal treatment intervals. In this study, we analyzed NVAMD patients whose treatment intervals had been optimized and stabilized to assess for any unique OCTA characteristics of CNV that may reflect treatment frequency and need.

## Methods

The AVATAR study is a single-site, prospective observational study investigating the role of OCTA in various retinal diseases. The study was approved by the Cleveland Clinic Institutional Review Board and adhered to all the tenets of the Declaration of Helsinki. All subjects provided written informed consent for participation in the study as approved by the IRB. Among subjects that participated in the AVATAR study, eyes that had undergone anti-VEGF therapy for NVAMD were identified. The diagnosis of NVAMD was based on clinical history and confirmatory diagnostic testing including spectral domain (SD) OCT, fluorescein angiography and indocyanine green angiography when necessary. Subjects with established specific treatment frequency of anti-VEGF therapy for at least 12 months were further identified and included in this study: (i) T&E requiring every 4–6 weeks dosing (TEq4-6w), (ii) T&E requiring every 7–12 weeks dosing (TEq7-12w), (iii) PRN not requiring injection for at least the last 12 months (PRN >12mo).

To establish treatment stability with anti-VEGF therapy, subjects were initially treated monthly until complete resolution of retinal fluid was achieved. Following fluid resolution, subjects were switched to a PRN approach where subjects were followed every 4–6 weeks for close evaluation of recurrence. If exudation recurred within 12 weeks of the last treatment, the subject was converted to a T&E protocol where eyes were treated every 4–6 weeks and then extended by 1–2 weeks once exudation had completely resolved or reached a stable state with a maximal response. If optimal anatomic outcomes were maintained, retreatment schedule was extended to a maximum interval of 12 weeks. Conversely, retreatment schedule was shortened by 1–2 weeks if retinal fluid increased on SD-OCT or new retinal hemorrhage was observed. The type of anti-VEGF agent (bevacizumab, ranibizumab or aflibercept) was determined by the physician; subjects were frequently treated with bevacizumab initially, and switching to an alternate anti-VEGF agent was considered if there was an insufficient response based on the resolution of fluid or durability of response.

For each subject, clinical demographics, past ocular histories and results of ocular examination during follow-up were collected and analyzed. Subjects with moderate or severe diabetic retinopathy or inability to obtain imaging on OCTA were excluded from the study. The number of intravitreal anti-VEGF injections administrated previous to OCTA visit, and visual acuity was collected. The type and any changes to the anti-VEGF agent used were not evaluated in this study. The Cirrus HD-OCT (Carl Zeiss Meditec, Germany) was utilized in all subjects to collect automatically generated OCT parameters such as central subfield thickness (the mean distance from internal limiting membrane to retinal pigment epithelium [RPE] in the central 1 mm subfield centered at the fovea) and cube average thickness, based on the 6 × 6 mm macula cube consisting 512 × 128 A-scans. The Cirrus HD-OCT was also used to classify neovascular lesions if they were located underneath the RPE (type 1), above the RPE (type 2), or intraretinally (type 3 neovascularization, the diagnosis was confirmed on OCTA).

### OCT angiography image acquisition and analysis

OCTA was obtained once treatment stability, defined as no change in treatment interval during the last 12 months, was reached. For those eyes in TEq4-6w group, an OCTA imaging was obtained at a retreatment visit. For those eyes in TEq7-12w group, an OCTA imaging was obtained at a retreatment visit and 4 weeks after the injection at a non-treatment visit. This allowed for the assessment of CNV flow dynamics that might be different in the following conditions: 1) 4 weeks after treatment between TEq4-6w and TEq7-12w group, 2) retreatment visit and 4 weeks after the treatment in TEq7-12w group.

OCTA volumes of 3 × 3 mm and 6 × 6 mm were obtained using the RTVue XR spectral domain OCT device (Optovue Inc., Freemont, CA). At a rate of 70,000 A-scans per second, the device produced two OCT volumes consisting of 304 × 304 A-scans each in approximately 2.6 seconds. A split-spectrum amplitude decorrelation angiography (SSADA) algorithm was applied to produce OCTA imaging. The AngioVue OCTA software was used to visualize the region of interest. The boundary for the RPE and Bruch’s membrane was fully corrected manually (line-by-line validation for the whole macular cube). Using the en face OCTA images of outer retinal slab (outer plexiform layer to Bruch’s membrane) and choriocapillaris slab, the CNV were assessed by an experienced retina specialist in a non-blinded manner for quantitative and qualitative findings focusing on the following characteristics: CNV area, vessel density, CNV shape (circular or irregular), CNV location (foveal involving or foveal sparing), the presence of core vessels, the presence of margin loops (small loops and/or large loops) and CNV maturity [27, 28]. The presence and depth of CNV relative to RPE was confirmed by decorrelation overlay on B-scan images. CNV core vessels were defined as a vessel of the greater caliber (with minimal caliber set at 100 μm in this study) that branch off smaller vessels [27]. CNV maturity was graded as immature form (presence of a rosette or tangle of indistinct vessels), mature form (presence of a distinct large dilated core vessel), or hyper mature form (presence of “dead tree” morphology or large, straight and dilated filamentous vessels) [28].

**Fig 1:**
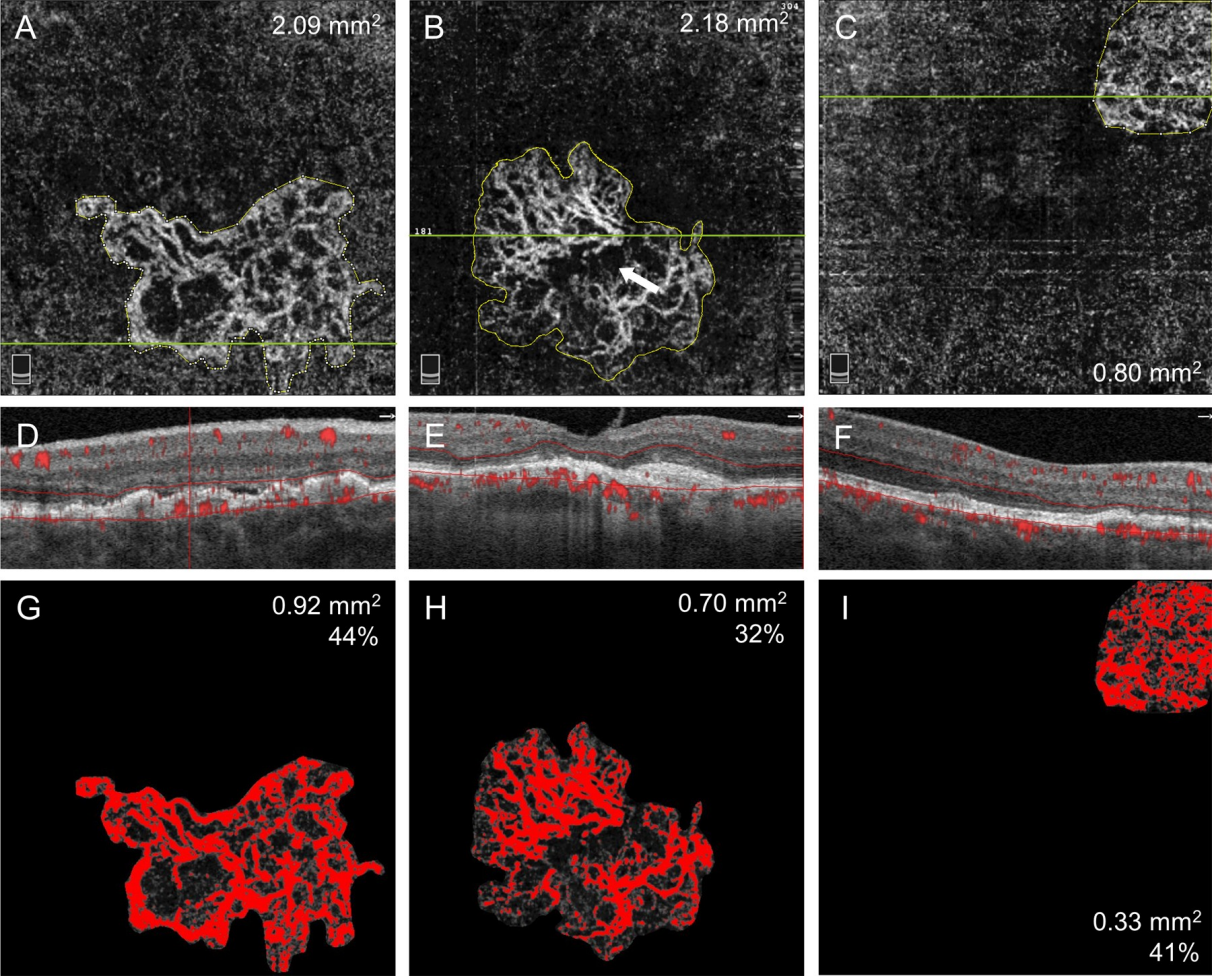
Representative cases in each treatment group demonstrating optical coherence tomography angiography (OCTA) of choroidal neovascularization (CNV) in neovascular age-related macular degeneration. (**Left column**) The right eye of a 73-year-old male in TEq4-6w group receiving anti-vascular endothelial growth factor (VEGF) therapy with 5 weeks interval (post 77 injections) for type 1+2 CNV (type 2 component is not visualized). CNV morphology is classified as irregular in shape, foveal involving, core vessel present, large loops, and mature form. CNV area is 2.09 mm^2^ and vessel density is 44%. (**Middle column**) The right eye of an 83-year-old male in TEq7-12w group receiving anti-VEGF therapy with 11 weeks interval (post 21 injections) for type 1+2 CNV. CNV morphology is classified as irregular in shape, foveal involving, core vessel absent, small and large loops, and mature form. CNV area is 2.18 mm^2^ and vessel density is 32%. (**Right column**) The right eye of a 75-year-old male in PRN>12mo group who had not received anti-VEGF therapy for 38 months after 13 injections for type 1 CNV. CNV morphology is classified as circular in shape, foveal sparing, core vessel absent, small loops, and mature form. CNV area is 0.80 mm^2^ and vessel density is 41%. **A-C,** En face OCTA image (3 × 3 mm) of outer retinal slab demonstrate well circumscribed neovascular complex. The manually depicted margin of the neovascular complex is shown in yellow line with CNV area. **D-F,** OCT B-scans showing fibrovascular retinal pigment epithelium detachment with vascular flow overlay. **G-I,** Binary processed image of the neovascular complex using Otsu algorism. Vessel flow area and vessel density are shown.

CNV area and vessel density were analyzed on en face OCTA images using Image J software Version 1.50i (National Institutes of Health, Bethesda, MD). To retain consistency, only 3 × 3 mm scan was used for this particular analysis. The border of the vascular complex in the outer retinal slab was defined manually as shown in Fig 1, then the area of neovascular membrane was estimated using the following equation: CNV area (mm^2^) = CNV area (pixel) × (3 mm/304 pixel)^2^. Otsu algorism was applied for binary image processing, and vessel density was calculated as a percentage of area occupied by the vessels [23, 29]. Vessel density was not calculated for small CNV (i.e., CNV area less than 0.1 mm^2^) since the value may potentially be inaccurate or biased.

**Fig 2:**
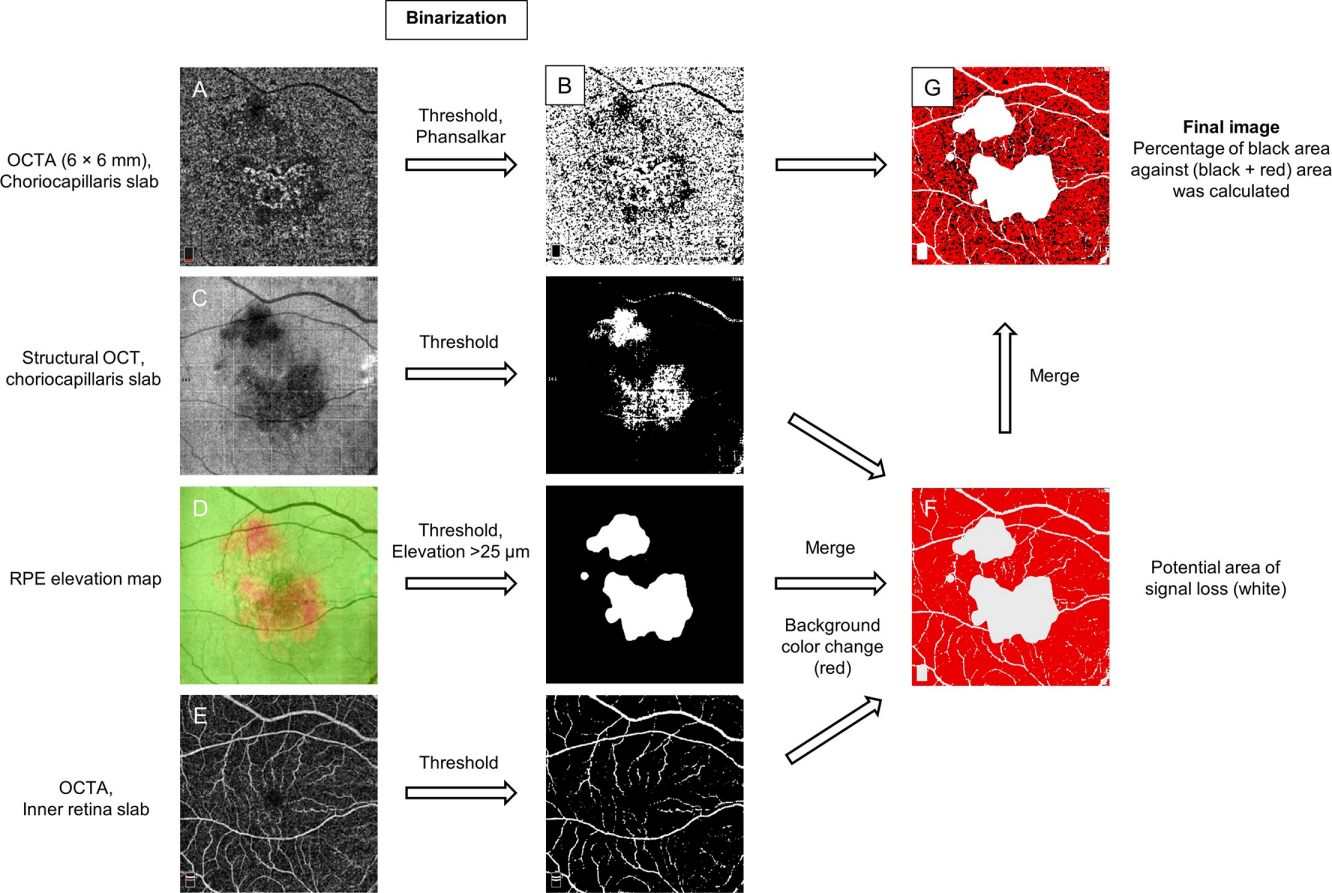
The process of quantifying the percentage of choriocapillaris area of nonperfusion (PCAN) or flow void in eyes with neovascular age-related macular degeneration. Using Image J software, the en face OCT angiography (OCTA) of the choriocapillaris slab (**A**) is binarized using “Phansalkar” algorithm (**B**), black area illustrate the flow void. En face structural OCT of the choriocapillaris slab (**C**), the RPE elevation map (**D**), OCTA of the inner retinal slab (**E**) are all binarized using a specific algorithm and merged supposedly to illustrate the potential area of signal loss at the level of choriocapillaris (**F**). Subsequently, two images (**B** and **F**) are merged to create the final image (**G**). PCAN is calculated as the percentage of the black area against (black + red) area.

Percentage of choriocapillaris area of nonperfusion (PCAN) or flow void on OCTA was measured also using Image J with an aim to estimate the loss or hypo-perfusion of the choriocapillaris. Only 6 × 6 mm scan with sufficient image quality (scan quality 5/10 or over) was used for this analysis. En face OCTA of choriocapillaris slab with 10 μm set at 5–20 μm below the Bruch’s membrane was binarized using “Phansalkar” algorithm with the radius setting at 0 pixels. The potential area of signal loss was determined using the following methods similar to previous reports [30, 31] (Fig 2); (a) 8-bit grayscale en face structural OCT of the choriocapillaris slab was binarized with threshold algorithm (0 to 63 = white, 64 to 255 = black) to highlight the dark area that could lead to false positive hypoperfusion, (b) en face RPE elevation map was binarized with color threshold algorithm to delineate the area of RPE elevation of more than 25 μm where may be subject to the loss of signal transmission, (c) 8-bit grayscale en face OCTA of inner retinal slab was binarized with threshold algorithm (0 to 127 = white, 128 to 255 = black) to exclude projection artifacts due to inner retinal vessels. All the images were then merged to create the final image, and the percentage of choriocapillaris area with flow void or PCAN was calculated as the percentage of black area (flow void) against (black + red) area. For this particular analysis, the fellow eye of study subjects with unilateral NVAMD was also analyzed.

### Statistical analysis

Data analyses were performed using R software version 3.2.3 (Software Foundation's GNU project, https://www.r-project.org/). Patient clinical demographics and main results are expressed as the mean ± standard deviation or median (interquartile range [IQR]). Both parametric and nonparametric statistical tests were used. For continuous variables, the Kolmogorov-Smirnov test was used to test the normal distribution, and the Bartlett test was used to compare the variance of three groups. Fisher’s exact test was performed for categorical variables. Man-Whitney U test was applied for quantitative nonparametric data comparing two groups. One-way analysis of variance (ANOVA) or Kruskal-Wallis test was used for quantitative parametric or nonparametric data among three groups, with Bonferroni correction for multiple comparisons when appropriate. For correlation analysis, nonparametric Spearman's test was used to calculate correlation coefficients. All probabilities were two-tailed, and the statistical significance level was set at P < .05. The dataset can be found as a supplementary table (S1 Table).

## Results

The clinical characteristics of the patients are summarized in Table 1. The study included 40 eyes of 31 patients (13 female patients, 18 male patients), with a mean age of 79.9 ± 6.2 (range 63–93) years at imaging. The median Snellen visual acuity was 20/40 (IQR 20/25-20/50, median logMAR VA: 0.30, IQR 0.10–0.40), the median central subfield thickness was 248 μm (IQR 225 to 271), the median cube average thickness was 269 μm (IQR 254 to 280). The TEq4-6w group exhibited the greatest median central subfield thickness (270 μm, P < .004). The classification of CNV was Type 1 (without type 2 component) in 27 (67.5%) eyes, Type 1+2 in 11 (27.5%) eyes and Type 3 neovascularization in 2 (5%) eyes. There was no difference in CNV classification between the three treatment groups. There was a statistically significant difference in the median number of anti-VEGF injections within the last 12 months follow-up (P < .001), and in the median total number of previous anti-VEGF injections (P < .001) among the three groups; The PRN>12mo group had received the least total number of anti-VEGF injections (median 5.5, IQR 4–7). OCTA allowed the identification of neovascular complex in 34 (85%) eyes, and the number of eyes whose CNV was identified on OCTA in TEq4-6w, TEq7-12w and PRN>12mo group was 17 (81%), 10 (91%) and 7 (88%) eyes, respectively.

**Table 1:** Patients demographics.

Demographics	TEq4-6w *n* = 21	TEq7-12w *n* = 11	PRN>12mo *n* = 8	All *n* = 40	P value
Age, years, mean ± SD	78.8 ± 6.4	79.1 ± 4.3	84.1 ± 6.7	79.9 ± 6.2	.097^a^
BCVA (log MAR), median (IQR)	0.30 (0.10–0.48)	0.30 (0.18–0.35)	0.14 (0.07–0.33)	0.30 (0.10–0.40)	.50^b^
Central subfield thickness, μm, median (IQR)	270 (247–316)	233 (219–242)	230 (214–255)	248 (225–271)	.004^b^
Cube average thickness, μm, median (IQR)	268 (258–278)	260 (251–283)	272 (258–280)	269 (254–280)	.85^b^
CNV type, *n* (%)					
Type 1	12 (57%)	8 (73%)	7 (87.5%)	27 (67.5%)	.060^c^
Type 1+2	9 (43%)	2 (18%)	0	11 (27.5%)	
Type 3 neovascularization	0	1 (9%)	1 (12.5%)	2 (5%)	
Number of anti-VEGF injections within 12 months, *n*, median (IQR)	9 (8–10)	6 (4.5–6.5)	0	7 (4–9)	< .001^b^
Total number of anti-VEGF injections, *n*, median (IQR)	29 (14–36)	27 (19.5–34)	5.5 (4–7)	22 (8–34)	< .001^b^
CNV identified on OCTA, *n* (%)	17 (81%)	10 (91%)	7 (88%)	34 (85%)	.85^c^

BCVA = best corrected visual acuity; CNV = choroidal neovascularization; IQR = interquartile range; OCTA = optical coherence tomography angiography; PRN = Pro re nata (treat as needed); SD = standard deviation; TE = treat and extend.

^a^ P value calculated using the one-way analysis of variance.

^b^ P value calculated using the Kruskal-Wallis test.

^c^ P value calculated using the Fisher’s exact test.

### OCTA findings

**Fig 3:**
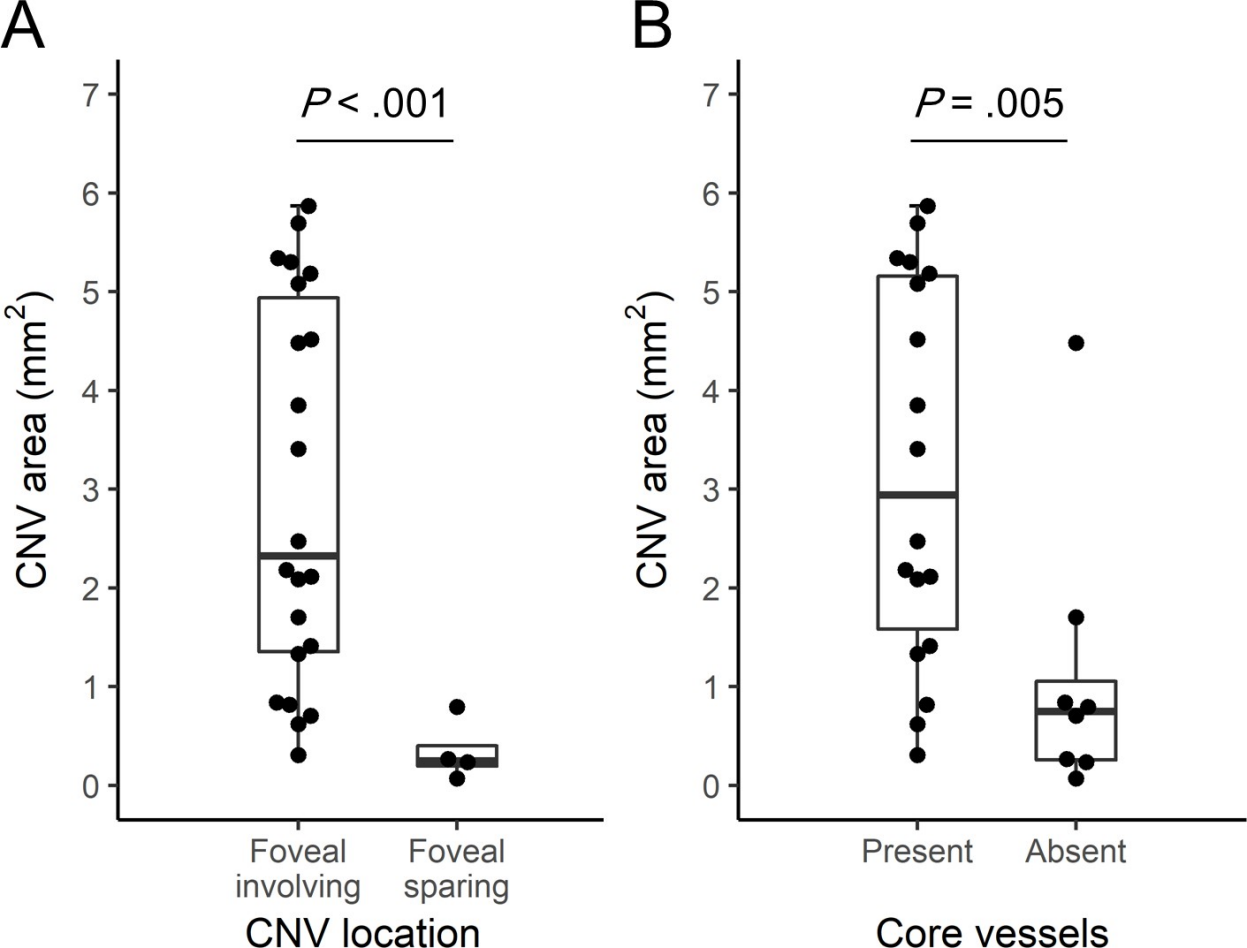
The area of choroidal neovascularization (CNV) on en face OCT angiography by CNV location and the presence of core vessels in all study eyes. **A,** A boxplot showing the area of CNV based on the CNV location. Each dot represents one eye. **B,** A boxplot showing the area of CNV based on the presence of core vessels.

A total of 29 (73%) eyes were further analyzed for the morphology of CNV; 5 eyes whose CNV was identified on OCTA (confirmed by abnormal decorrelation overlay on B-scan) but with indistinct vessel morphology were excluded in this analysis. The characteristics of CNV on OCTA by analysis group are shown in Table 2 (representative cases are shown in Fig 1). The median CNV area in TEq4-6w, TEq7-12w and PRN>12mo group was 2.12 mm^2^ (IQR, 0.83 to 4.18), 2.47 mm^2^ (IQR, 1.71 to 5.08) and 0.53 mm^2^ (IQR, 0.24 to 1.26) (P *=* .14), and the mean vessel density was 38%, 36% and 42% (P *=* .19), respectively. In qualitative analysis, a statistical difference was found in CNV shape (P *=* .012), and CNV location (P *=* .003); 90% of CNVs in TEq7-12w group were irregular in shape involving foveal center, while 67% of CNVs in PRN>12mo group were circular in shape sparing foveal center. No statistical difference was found in the presence of core vessels (P *=* .23), the presence of small margin loops (P *=* .20), large margin loops (P *=* .14), or CNV maturity (P *=* .40) among three groups. The mature form was most prevalent in all three groups comprising 54 to 83% of eyes, and hyper mature form was only observed in TEq4-6w and TEq7-12w group comprising approximately 30%. CNV area based on the location and the presence of core vessels is shown in Fig 3. In all study eyes, CNV area was significantly larger when CNV involved the foveal center (median 2.18 mm^2^ vs 0.25 mm^2^, P < .001) and core vessels were present (median 2.94 mm^2^ vs 0.71 mm^2^, P = .005).

**Table 2:** Characteristics of CNV on OCTA by analysis group.

CNV Measures on OCTA	TEq4-6w *n* = 13	TEq7-12w *n* = 10	PRN>12mo *n* = 6	P value
CNV area, mm^2^, median (IQR)	2.12 (0.83–4.18)	2.47 (1.71–5.08)	0.53 (0.24–1.26)	.14^a^
Vessel density, %, mean ± SD	38 ± 5	36 ± 8	42 ± 4	.19^b^
CNV shape, *n* (%)				
Circular	5 (38.5%)	1 (10%)	5 (83%)	.012^c^
Irregular	8 (61.5%)	9 (90%)	1 (17%)	
CNV location, *n* (%)				
Foveal involving	12 (92%)	10 (100%)	2 (33%)	.003^c^
Foveal sparing	1 (8%)	0	4 (67%)	
Presence of core vessels, *n* (%)				
Present	10 (77%)	6 (60%)	2 (33%)	.23^c^
Absent	3 (23%)	4 (40%)	4 (67%)	
Presence of margin loops, *n* (%)				
Small loops	7 (54%)	9 (90%)	4 (67%)	.20^c^
Large loops	6 (46%)	7 (70%)	1 (17%)	.14^c^
CNV Maturity, *n* (%)				
Immature form	2 (15%)	0	1 (17%)	.40^c^
Mature form	7 (54%)	7 (70%)	5 (83%)	
Hyper mature form	4 (31%)	3 (30%)	0	

CNV = choroidal neovascularization; IQR = interquartile range; OCTA = optical coherence tomography angiography; PRN = Pro re nata (treat as needed); SD = standard deviation; TE = treat and extend.

Results are expressed as a mean ± standard deviation.

^a^ P value calculated using the Kruskal-Wallis test.

^b^ P value calculated using the one-way analysis of variance.

^c^ P value calculated using the Fisher’s exact test.

**Fig 4:**
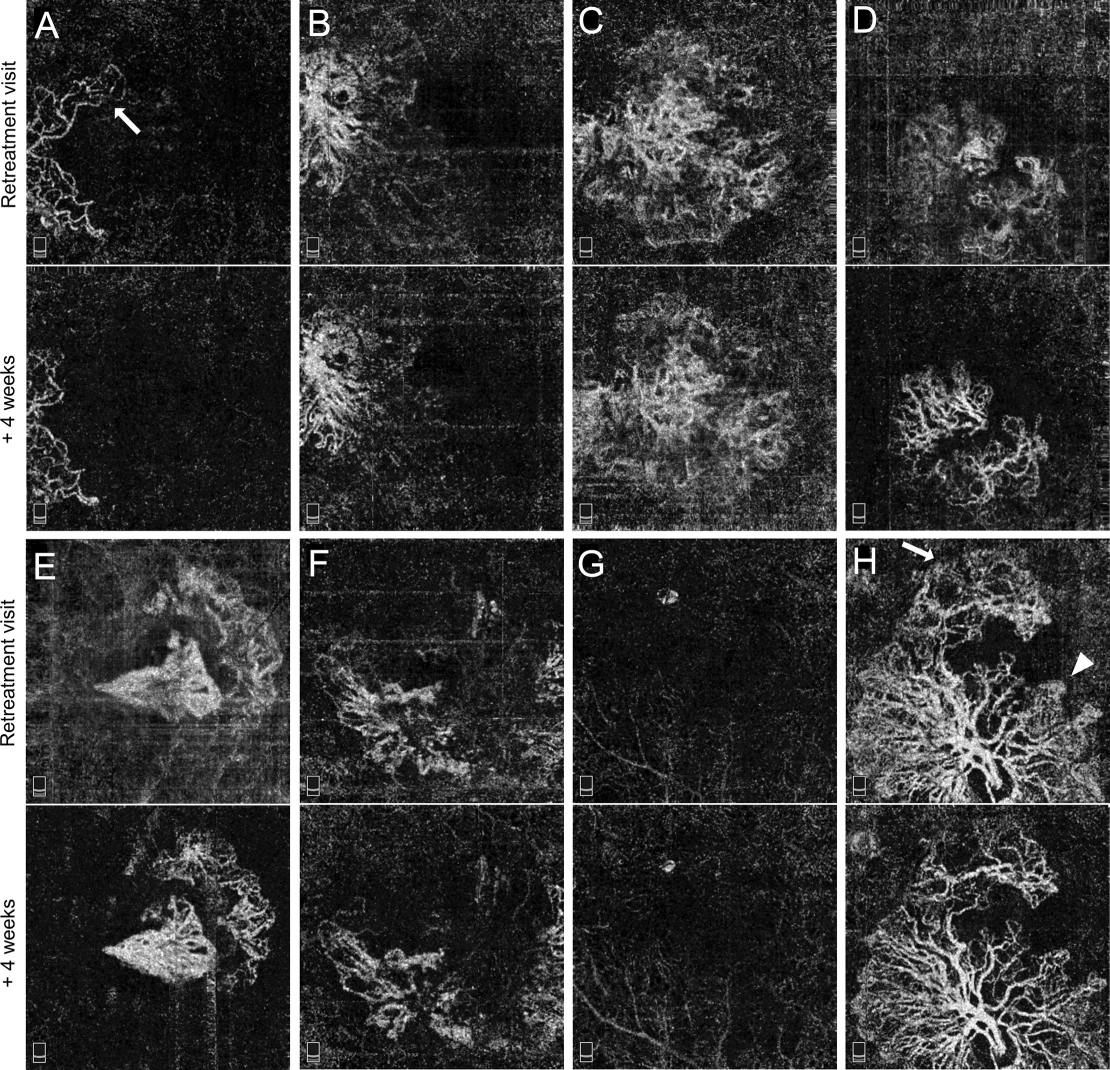
Comparison in choroidal neovascularization (CNV) appearance on OCT angiography (OCTA) (3 × 3 mm, outer retina slab) observed at retreatment visit and 4 weeks after the treatment in TEq7-12w group. **A-H,** Eight eyes with analyzable OCTA at both retreatment visit and 4 weeks after that visit. Some of the vascular components are only visible at retreatment visit (arrow), and a slight dilation of microvasculature is observed at retreatment visit. In two eyes (**A** and **C**), the location of CNV was graded as foveal involving due to the presence of pigment epithelial detachment with possible inactive fibrovascular components on B-scan crossing the foveal center.

Eyes in TEq7-12w group were further evaluated for any differences in CNV morphological characteristics on OCTA observed at retreatment visit and 4 weeks after that treatment. Among 10 eyes in TEq7-12w group, 8 eyes had analyzable OCTA obtained 4 weeks apart as shown in Fig 4. Two eyes were unable to evaluate; 1 eye due to the lack of OCTA images at retreatment visit and 1 eye due to insufficient image quality. Overall, there was a subtle difference in en face OCTA between the 2 visits; some vascular components were only visible at retreatment visit (arrow) and a slight dilation of microvasculature was observed at retreatment visit (arrowhead). However, these differences did not affect the qualitative assessment on the characteristics of CNV as described in Table 2.

**Fig 5:**
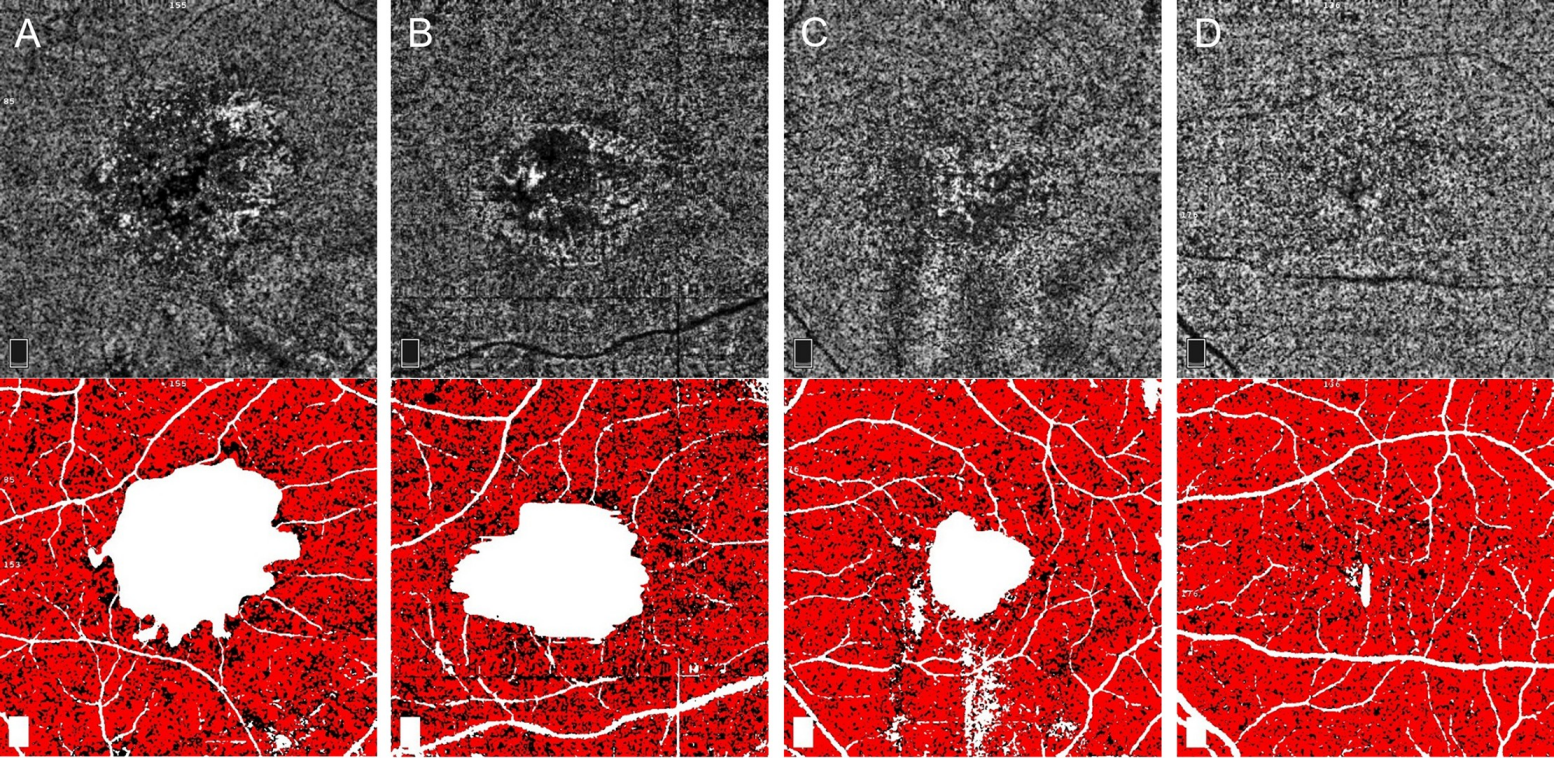
**Representative cases illustrating en face OCT angiography of the choriocapillaris slab (top row) and the final image created to calculate the percentage of choriocapillaris area of nonperfusion (PCAN) (bottom row) in each group. A,** Right eye of a 73-year-old male in TEq4-6w group with type 1+2 choroidal neovascularization (CNV). PCAN is 26%. **B,** Right eye of an 82-year-old male in TEq7-12w group with type 1 CNV. PCAN is 33%. **C,** Left eye of an 80-year-old male in PRN>12mo group with type 1 CNV. PCAN is 19%. **D,** Left eye of a 79-year-old male in the fellow-eye group without a clinical diagnosis of CNV. PCAN is 15%.

**Fig 6:**
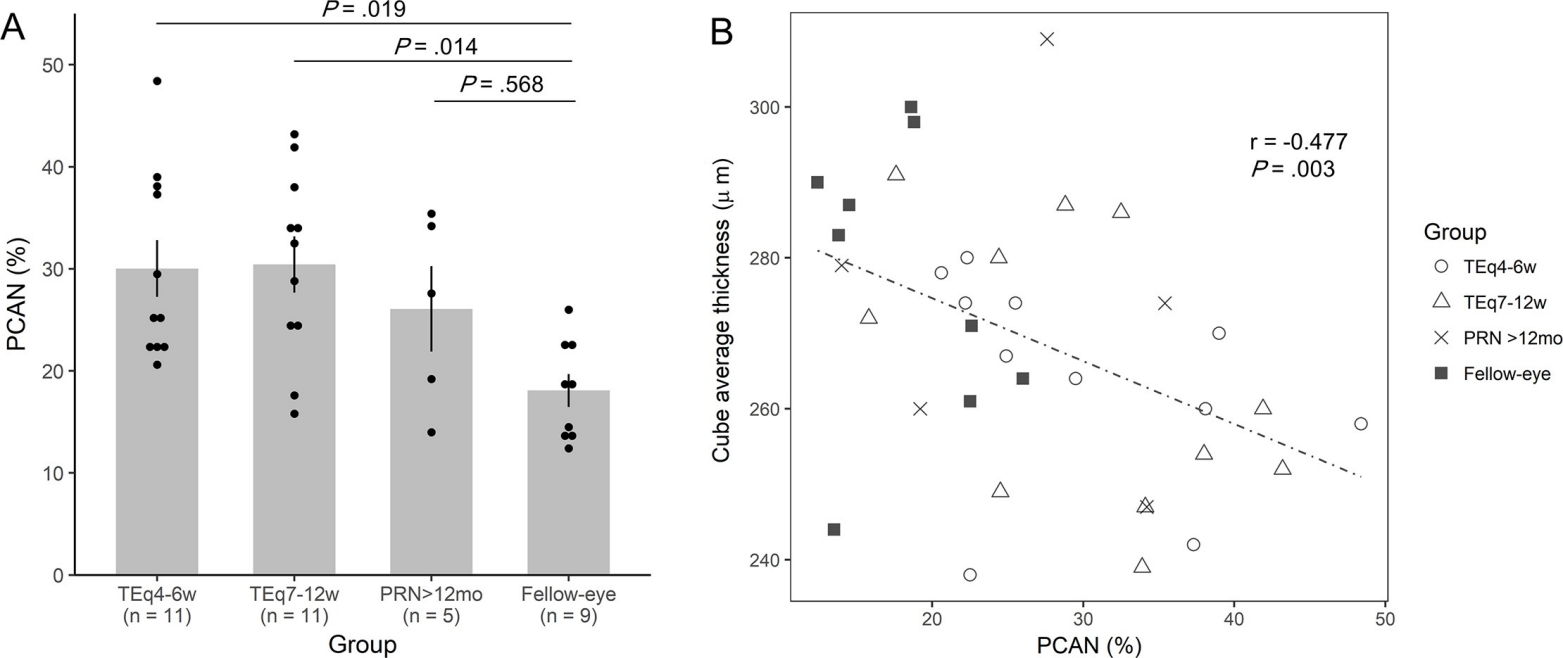
Percentage of choriocapillaris area of nonperfusion (PCAN) in each group and its correlation with cube average thickness. **A,** A bar plot representing mean PCAN in each group. Error bars represent standard error of the mean and each dot represents one eye. Mean PCAN was significantly higher in TEq4-6w and TE7-12w group compared with the fellow-eye group. **B,** Scatter plot representing PCAN versus cube average thickness showing a negative correlation between two variables in all study eyes.

In the choriocapillaris flow void analysis, a total of 27 (60%) eyes were included; 5 eyes were excluded due to the lack of 6 × 6 mm OCTA image, and 8 eyes were excluded due to the insufficient image quality of the OCTA (representative cases are shown in Fig 5). Also, a total of 9 fellow eyes of study subjects with unilateral NVAMD were included as a fellow-eye group. The results of the choriocapillaris flow void are shown in Fig 6A. The mean PCAN in TEq4-6w, TEq7-12w, PRN>12mo group was 30%, 30%, and 26%, respectively, which was not statistically significant across three treatment groups (P *=* .66). However, the mean PCAN was significantly higher in TEq4-6w (P *=* .019) and TEq7-12w (P *=* .014) group compared with 18% in the fellow-eye group. Also, correlation analysis revealed that PCAN was negatively correlated with macular cube average thickness in all study eyes (r = -0.477, P *=* .003) (Fig 6B).

## Discussion

Recent studies have shown that OCTA enables noninvasive and rapid visualization of neovascular membranes in great morphologic detail, allowing the qualitative and quantitative evaluation of their microvascular features in both exudative (active) and nonexudative (inactive) CNVs [27]. However, not all CNVs are detectable with OCTA, due in part to the current limitations of the OCTA technology, and by factors such as the activity of NVAMD, history of anti-VEGF therapy, or type of neovascularization. In this study, CNV was identified on OCTA in 85% of eyes (vessel morphology assessment was feasible in 73% of eyes) which was in reasonable accordance with previous reports that identified CNV in 34–100% of eyes including clinically active NVAMD [26, 32–34], and in 100% of eyes with clinically inactive NVAMD not requiring any treatment over 6 months [23].

Fine capillary vessels of the neovascular complex in treatment naïve eyes are reported to significantly reduce its density after anti-VEGF therapy [35], while vessel density in a chronic setting following multiple injections becomes less responsive after each treatment [26]. In the present study, the mean vessel density was comparable between TEq4-6w group at 38% and TEq7-12w group at 36%, which was slightly lower than PRN>12mo group at 42%. Plausible explanations include the decreased vascular activity in eyes with TEq4-6w and TEq7-12w group due to ongoing suppression of intraocular VEGF levels. It may also be due to the limitations of the current OCTA device, incapable of detecting very slow blood flow [36, 37]. The capillary vessel flow is presumed to be in the recovery phase in TEq4-6w and TEq7-12w group, and the flow speed might have been below a detectable level, leading to underestimation of vessel density. The size of the CNV may also have impacted the vessel density. In a large CNV, the internal lesion may appear hyporeflective due to either actual paucity of the vascular component along with fibrosis development, or OCT signal attenuation (Fig 1B, arrow). Since the CNV size was relatively large in TEq4-6w and TEq7-12w group, overall vessel density may have been underestimated.

There was no difference in CNV area across three groups (P *=* .14). Nevertheless, in PRN>12mo group, 67% of eyes exhibited the identical OCTA characteristics of CNV including the area of less than 1 mm^2^, foveal sparing and the absence of core vessels after a relatively small total number of anti-VEGF injections (median 6, range 3–13). This combination of characteristics might represent one aspect of clinically inactive CNV. Of note, CNV area was significantly larger when CNV involved the foveal center and core vessel was present in all study eyes (Fig 3). The development of core vessels is considered to reflect the maturation of the neovascular membrane [18, 27], and it has been suggested that the absence of core vessels may be a protective factor against the increased activity of CNV [27]. The present findings might indicate that CNV sparing foveal center with the absence of core vessel have a chance of becoming an inactive state without exudation for an extended period. Meanwhile, there were minimal distinguishing characteristics between those eyes that required ongoing therapy with T&E regimen, which was in line with the previous report by Roberts et al where authors evaluated 25 eyes with NVAMD for qualitative and quantitative features on en face OCTA images between good responders treated less frequently than 6 weeks versus poor responders treated every 6 weeks or more frequently and found no association in CNV features [38]. Recently, Nesper et al retrospectively explored the 3 dimensional complexity of CNV lesions in 51 eyes with NVAMD and found that poor responders treated more frequently than 6 weeks intervals had a greater number of CNV flow layers and greater CNV flow thickness [39]. Their study results may suggest that CNV with more complex 3 dimensional structure are associated with more frequent treatment and 2 dimensional en face OCTA analysis may be less helpful for understanding the treatment requirement in eyes with long-standing NVAMD.

Quantitative analysis of choriocapillaris microvascular flow on OCTA requires meticulous attention as choriocapillaris slab is susceptible to artifacts, such as motion and projection artifacts. It becomes even more challenging in eyes with neovascular AMD, as pigment epithelial detachment frequently induce segmentation error of Bruch’s membrane plane and OCT signal loss at the level of the choriocapillaris. In the present study, we performed complete manual line-by-line validation of RPE and Bruch’s membrane band of each OCTA scan to eliminate the possibility of segmentation error. When analyzing PCAN, we employed various slabs and utilized specific binary algorithms to create a composite image to locate the area with potential artifacts and removed from the analysis.

We could not find any statistical differences in PCAN among the three treatment groups. However, PCAN was significantly greater in TEq4-6w and TEq7-12w group compared with the fellow-eye group without NVAMD which was consistent with recent reports. Treister et al investigated 34 eyes with exudative AMD and revealed that the area of choriocapillaris flow void was greater in eyes with exudative AMD compared with the fellow eye with treatment-naïve subclinical CNV [30]. Borrelli et al studied 21 eyes with unilateral type 3 neovascularization and demonstrated that the percent of nonperfused choriocapillaris area was greater compared with the fellow eyes [31]. Previous histological studies have shown that the loss of choriocapillaris precede the development of CNV in eyes with NVAMD [40], and type 1 CNV complex has been hypothesized to become a source of continuous nourishment for RPE and photoreceptors, playing a role as compensatory vessels of choriocapillaris that may have a protective effect in the prevention of RPE atrophy [41, 42]. In the present study, a negative correlation was found between PCAN and macular cube average thickness in all study subjects, indicating that choriocapillaris flow void was more severe in eyes with thinner retina. The pathomechanism of this correlation is beyond the scope of this study. Perhaps, reduced cube average thickness in some cases might be attributed to impaired perfusion of choriocapillaris with insufficient oxygen supply to the RPE or outer retina causing loss of retinal cells. Further study is warranted to elucidate the role of choriocapillaris flow void in the pathogenesis and management of NVAMD.

Our study has several impotant limitations that should be considered. Regarding the study design, the definition of treatment groups based on 1 year of treatment stability and the inclusion of eyes with large disparities in treatment history may have confounded the results. It is possible that CNV membrane evolved through multi-year treatment may be unable to predict treatment burden or prognosis by relying purely on 2-dimensional en face morphological features. As for the quantitative and qualitative analysis of OCTA imaging, observer-expectancy bias may have been introduced because OCTA was assessed in a non-blinded manner. Our study is also limited by small sample size including all types of CNV. The use of swept source OCTA may have better delineated the existence and morphology of CNV, as prior studies demonstrated that CNV size determined by swept-source OCTA was larger than that of spectral domain OCTA used in this study [43, 44]. Moreover, CNV size and morphology were analyzed in two dimensions en face image in this study. The volumetric analysis of CNV may better reflect the actual characteristics of CNV. Furthermore, we did not analyze the type of anti-VEGF agents used within each group because the selection and switching of the agents largely relied on physician’s discretion and was not randomized. A small difference in the duration of action could have affected treatment interval thereby confusing overall results. Further study is warranted to clarify the individual influence of each agent on CNV morphology observed with OCTA. Lastly, the area of potential signal loss (e.g., RPE elevation) was excluded when analyzing PCAN. A severe loss of choriocapillaris might exist under the multilayered neovascular complex. Therefore, PCAN in our study may not represent the actual severity of choriocapillaris loss.

In summary, OCTA successfully imaged CNV in most eyes undergoing anti-VEGF therapy for CNV. There were limited distinguishing characteristics between those eyes that required ongoing therapy with T&E regimen. This suggests visualization of CNV on OCTA alone might be insufficient to determine the short-lasting treatment requirement with the technology evaluated in this study. However, the combination of CNV sparing foveal center with the absence of the core vessels might represent one aspect of clinically inactive CNV. More research is needed to identify specific CNV characteristics on OCTA that may become a useful tool for the prediction of treatment intervals.

## Supporting information

S1 Table Table including the dataset available as S1 Table. (XLSX)

## Abbreviations

ANOVA: one-way analysis of variance
CNV: choroidal neovascularization
IQR: interquartile range
NVAMD: neovascular age-related macular degeneration
OCTA: optical coherence tomography angiography
PCAN: percentage of choriocapillaris area with flow void
PRN: pro re nata (“treat as-needed”)
SD-OCT: spectral domain optical coherence tomography
T&E: treat-and-extend
VA: visual acuity
VEGF: vascular endothelial growth factor

## References

[1] Bourne RR, Jonas JB, Flaxman SR, Keeffe J, Leasher J, Naidoo K et al Prevalence and causes of vision loss in high-income countries and in Eastern and Central Europe: 1990–2010. Br J Ophthalmol. 2014;5:629–638. 10.1136/bjophthalmol-2013-304033 24665132

[2] Rosenfeld PJ, Brown DM, Heier JS, Boyer DS, Kaiser PK, Chung CY et al Ranibizumab for neovascular age-related macular degeneration. N Engl J Med. 2006;14:1419–1431. 10.1056/NEJMoa054481 17021318

[3] Martin DF, Maguire MG, Ying GS, Grunwald JE, Fine SL, Jaffe GJ. Ranibizumab and bevacizumab for neovascular age-related macular degeneration. N Engl J Med. 2011;20:1897–1908. 10.1056/NEJMoa1102673 PMC3157322 21526923

[4] Heier JS, Brown DM, Chong V, Korobelnik JF, Kaiser PK, Nguyen QD et al Intravitreal aflibercept (VEGF trap-eye) in wet age-related macular degeneration. Ophthalmology. 2012;12:2537–2548. 10.1016/j.ophtha.2012.09.006 23084240

[5] Rofagha S, Bhisitkul RB, Boyer DS, Sadda SR, Zhang K. Seven-year outcomes in ranibizumab-treated patients in ANCHOR, MARINA, and HORIZON: a multicenter cohort study (SEVEN-UP). Ophthalmology. 2013;11:2292–2299. 10.1016/j.ophtha.2013.03.046 23642856

[6] Krebs I, Glittenberg C, Ansari-Shahrezaei S, Hagen S, Steiner I, Binder S. Non-responders to treatment with antagonists of vascular endothelial growth factor in age-related macular degeneration. Br J Ophthalmol. 2013;11:1443–1446. 10.1136/bjophthalmol-2013-303513 23966368

[7] Gillies MC, Campain A, Barthelmes D, Simpson JM, Arnold JJ, Guymer RH et al Long-term outcomes of treatment of neovascular age-related macular degeneration: Data from an observational study. Ophthalmology. 2015;9:1837–1845. 10.1016/j.ophtha.2015.05.010 26096346

[8] Rezaei KA, Stone, T.W. Global trends in retina. American Society of Retina Specialists. 2015. Available from: https://www.asrs.org/content/documents/2015_global_trends_in_retina_survey_-_for_website.pdf.

[9] Fung AE, Lalwani GA, Rosenfeld PJ, Dubovy SR, Michels S, Feuer WJ et al An optical coherence tomography-guided, variable dosing regimen with intravitreal ranibizumab (Lucentis) for neovascular age-related macular degeneration. Am J Ophthalmol. 2007;4:566–583. 10.1016/j.ajo.2007.01.028 17386270

[10] Gupta OP, Shienbaum G, Patel AH, Fecarotta C, Kaiser RS, Regillo CD. A treat and extend regimen using ranibizumab for neovascular age-related macular degeneration clinical and economic impact. Ophthalmology. 2010;11:2134–2140. 10.1016/j.ophtha.2010.02.032 20591490

[11] Boyer DS, Antoszyk AN, Awh CC, Bhisitkul RB, Shapiro H, Acharya NR. Subgroup analysis of the MARINA study of ranibizumab in neovascular age-related macular degeneration. Ophthalmology. 2007;2:246–252. 10.1016/j.ophtha.2006.10.045 17270674

[12] Smailhodzic D, Muether PS, Chen J, Kwestro A, Zhang AY, Omar A et al Cumulative effect of risk alleles in CFH, ARMS2, and VEGFA on the response to ranibizumab treatment in age-related macular degeneration. Ophthalmology. 2012;11:2304–2311. 10.1016/j.ophtha.2012.05.040 22840423

[13] Kloeckener-Gruissem B, Barthelmes D, Labs S, Schindler C, Kurz-Levin M, Michels S et al Genetic association with response to intravitreal ranibizumab in patients with neovascular AMD. Invest Ophthalmol Vis Sci. 2011;7:4694–4702. 10.1167/iovs.10-6080 21282580

[14] Heier JS, Boyer D, Nguyen QD, Marcus D, Roth DB, Yancopoulos G et al The 1-year results of CLEAR-IT 2, a phase 2 study of vascular endothelial growth factor trap-eye dosed as-needed after 12-week fixed dosing. Ophthalmology. 2011;6:1098–1106. 10.1016/j.ophtha.2011.03.020 21640258

[15] Schaal S, Kaplan HJ, Tezel TH. Is there tachyphylaxis to intravitreal anti-vascular endothelial growth factor pharmacotherapy in age-related macular degeneration? Ophthalmology. 2008;12:2199–2205. 10.1016/j.ophtha.2008.07.007 18930553

[16] Eghoj MS, Sorensen TL. Tachyphylaxis during treatment of exudative age-related macular degeneration with ranibizumab. Br J Ophthalmol. 2012;1:21–23. 10.1136/bjo.2011.203893 21733918

[17] Cho M, Barbazetto IA, Freund KB. Refractory neovascular age-related macular degeneration secondary to polypoidal choroidal vasculopathy. Am J Ophthalmol. 2009;1:70–78 e71. 10.1016/j.ajo.2009.02.012 19403115

[18] Spaide RF. Optical Coherence Tomography Angiography Signs of Vascular Abnormalization With Antiangiogenic Therapy for Choroidal Neovascularization. Am J Ophthalmol. 2015;1:6–16. 10.1016/j.ajo.2015.04.012 25887628

[19] Spaide RF. Rationale for combination therapy in age-related macular degeneration. Retina. 2009;6 Suppl:S5–7. 10.1097/IAE.0b013e3181ad237a 19553803

[20] Jia Y, Bailey ST, Wilson DJ, Tan O, Klein ML, Flaxel CJ et al Quantitative optical coherence tomography angiography of choroidal neovascularization in age-related macular degeneration. Ophthalmology. 2014;7:1435–1444. 10.1016/j.ophtha.2014.01.034 PMC4082740 24679442

[21] Coscas GJ, Lupidi M, Coscas F, Cagini C, Souied EH. Optical coherence tomography angiography versus traditional multimodal imaging in assessing the activity of exudative age-related macular degeneration: A new diagnostic challenge. Retina. 2015;11:2219–2228. 10.1097/IAE.0000000000000766 26398697

[22] Spaide RF, Klancnik JM Jr., Cooney MJ. Retinal vascular layers imaged by fluorescein angiography and optical coherence tomography angiography. JAMA Ophthalmol. 2015;1:45–50. 10.1001/jamaophthalmol.2014.3616 25317632

[23] Ichiyama Y, Sawada T, Ito Y, Kakinoki M, Ohji M. Optical Coherence Tomography Angiography Reveals Blood Flow in Choroidal Neovascular Membrane in Remission Phase of Neovascular Age-Related Macular Degeneration. Retina. 2017;4:724–730. 10.1097/IAE.0000000000001576 28248824

[24] Muakkassa NW, Chin AT, de Carlo T, Klein KA, Baumal CR, Witkin AJ et al Characterizing the Effect of Anti-Vascular Endothelial Growth Factor Therapy on Treatment-Naive Choroidal Neovascularization Using Optical Coherence Tomography Angiography. Retina. 2015;11:2252–2259. 10.1097/IAE.0000000000000836 26457400

[25] Tan AC, Dansingani KK, Yannuzzi LA, Sarraf D, Freund KB. Type 3 Neovascularization Imaged with Cross-Sectional and En Face Optical Coherence Tomography Angiography. Retina. 2017;2:234–246. 10.1097/IAE.0000000000001343 27749497

[26] Kuehlewein L, Bansal M, Lenis TL, Iafe NA, Sadda SR, Bonini Filho MA et al Optical Coherence Tomography Angiography of Type 1 Neovascularization in Age-Related Macular Degeneration. Am J Ophthalmol. 2015;4:739–748 e732. 10.1016/j.ajo.2015.06.030 26164826

[27] Carnevali A, Cicinelli MV, Capuano V, Corvi F, Mazzaferro A, Querques L et al Optical Coherence Tomography Angiography: A Useful Tool for Diagnosis of Treatment-Naive Quiescent Choroidal Neovascularization. Am J Ophthalmol. 2016:189–198. 10.1016/j.ajo.2016.06.042 27394033

[28] Xu D, Davila JP, Rahimi M, Rebhun CB, Alibhai AY, Waheed NK et al Long-term Progression of Type 1 Neovascularization in Age-related Macular Degeneration Using Optical Coherence Tomography Angiography. Am J Ophthalmol. 2018:10–20. 10.1016/j.ajo.2017.12.005 29269100

[29] Otsu N. A threshold selection method from gray-level histograms. IEEE Trans Syst Man Cybern. 1979;1:62–66.

[30] Treister AD, Nesper PL, Fayed AE, Gill MK, Mirza RG, Fawzi AA. Prevalence of Subclinical CNV and Choriocapillaris Nonperfusion in Fellow Eyes of Unilateral Exudative AMD on OCT Angiography. Transl Vis Sci Technol. 2018;5:19. 10.1167/tvst.7.5.19 PMC6166896 30280004

[31] Borrelli E, Souied EH, Freund KB, Querques G, Miere A, Gal-Or O et al Reduced Choriocapillaris Flow in Eyes with Type 3 Neovascularization and Age-Related Macular Degeneration. Retina. 2018;10:1968–1976. 10.1097/IAE.0000000000002198 29746411

[32] Kuehlewein L, Dansingani KK, de Carlo TE, Bonini Filho MA, Iafe NA, Lenis TL et al Optical Coherence Tomography Angiography of Type 3 Neovascularization Secondary to Age-Related Macular Degeneration. Retina. 2015;11:2229–2235. 10.1097/IAE.0000000000000835 26502007

[33] El Ameen A, Cohen SY, Semoun O, Miere A, Srour M, Quaranta-El Maftouhi M et al Type 2 Neovascularization Secondary to Age-Related Macular Degeneration Imaged by Optical Coherence Tomography Angiography. Retina. 2015;11:2212–2218. 10.1097/IAE.0000000000000773 26441269

[34] Miere A, Semoun O, Cohen SY, El Ameen A, Srour M, Jung C et al Optical Coherence Tomography Angiography Features of Subretinal Fibrosis in Age-Related Macular Degeneration. Retina. 2015;11:2275–2284. 10.1097/IAE.0000000000000819 26457397

[35] Kuehlewein L, Sadda SR, Sarraf D. OCT angiography and sequential quantitative analysis of type 2 neovascularization after ranibizumab therapy. Eye. 2015;7:932–935. 10.1038/eye.2015.80 PMC4506353 25976641

[36] de Carlo TE, Bonini Filho MA, Chin AT, Adhi M, Ferrara D, Baumal CR et al Spectral-domain optical coherence tomography angiography of choroidal neovascularization. Ophthalmology. 2015;6:1228–1238. 10.1016/j.ophtha.2015.01.029 25795476

[37] Spaide RF, Fujimoto JG, Waheed NK. Image Artifacts in Optical Coherence Tomography Angiography. Retina. 2015;11:2163–2180. 10.1097/IAE.0000000000000765 PMC4712934 26428607

[38] Roberts PK, Nesper PL, Gill MK, Fawzi AA. Semiautomated Quantitative Approach to Characterize Treatment Response in Neovascular Age-Related Macular Degeneration: A Real-World Study. Retina. 2017;8:1492–1498. 10.1097/IAE.0000000000001400 PMC5474212 27997513

[39] Nesper PL, Soetikno BT, Treister AD, Fawzi AA. Volume-Rendered Projection-Resolved OCT Angiography: 3D Lesion Complexity Is Associated With Therapy Response in Wet Age-Related Macular Degeneration. Invest Ophthalmol Vis Sci. 2018;5:1944–1952. 10.1167/iovs.17-23361 PMC5894925 29677356

[40] Biesemeier A, Taubitz T, Julien S, Yoeruek E, Schraermeyer U. Choriocapillaris breakdown precedes retinal degeneration in age-related macular degeneration. Neurobiol Aging. 2014;11:2562–2573. 10.1016/j.neurobiolaging.2014.05.003 24925811

[41] Grossniklaus HE, Green WR. Choroidal neovascularization. Am J Ophthalmol. 2004;3:496–503. 10.1016/j.ajo.2003.09.042 15013874

[42] Dansingani KK, Freund KB. Optical Coherence Tomography Angiography Reveals Mature, Tangled Vascular Networks in Eyes With Neovascular Age-Related Macular Degeneration Showing Resistance to Geographic Atrophy. Ophthalmic Surg Lasers Imaging Retina. 2015;9:907–912. 10.3928/23258160-20151008-02 26469229

[43] Novais EA, Adhi M, Moult EM, Louzada RN, Cole ED, Husvogt L et al Choroidal neovascularization analyzed on ultrahigh-speed swept-source optical coherence tomography angiography compared to spectral-domain optical coherence tomography angiography. Am J Ophthalmol. 2016:80–88. 10.1016/j.ajo.2016.01.011 PMC4811690 26851725

[44] Zhang Q, Chen CL, Chu Z, Zheng F, Miller A, Roisman L et al Automated Quantitation of Choroidal Neovascularization: A Comparison Study Between Spectral-Domain and Swept-Source OCT Angiograms. Invest Ophthalmol Vis Sci. 2017;3:1506–1513. 10.1167/iovs.16-20977 PMC5361585 28273317

